# Differences in substance use among trans, non-binary and cis people in Spain: a cross-sectional study

**DOI:** 10.1186/s13011-026-00741-6

**Published:** 2026-07-08

**Authors:** Florentino Quinteiro, Sara Moreno-García, María José Belza, Begoña Brime, Mark Padilla, Juan Miguel Guerras, Paule Gonzalez-Recio

**Affiliations:** 1 Preventive Medicine Department, Ramón y Cajal University Hospital, Madrid, Spain; 2 Mixed Research Institute ENS-UNED (IMIENS), Av Monforte de Lemos 5, Madrid, 28029 Spain; 3 National Health School, Carlos III Health Institute, Av Monforte de Lemos 5, Madrid, 28029 Spain; 4 Center for Biomedical Research Network in Epidemiology and Public Health (CIBERESP), Av Monforte de Lemos 5, Madrid, 28029 Spain; 5 Government Delegation for the National Drugs Plan, Ministry of Health, Madrid, Spain; 6 Research Center for Health and Society (REACH), Florida International University, Miami, FL USA; 7 National Epidemiology Center, Carlos III Health Institute, Madrid, Spain

**Keywords:** Trans, Non-binary, Substance use, Drugs, Cisgender comparison, Spain

## Abstract

**Background:**

Differences in substance use have been reported to be higher among transgender and non-binary people (TNBP) than among their cisgender peers, although previous comparison studies are limited, especially in Europe. This study aimed to estimate and compare past-year and past-month prevalence of multiple substances between TNBP and cis people in Spain.

**Methods:**

Cross-sectional study conducted with data from 19,948 individuals aged 15–45 years, 1582 of which were TNBP. Most TNBP were recruited through Transaludes survey, a national self-administered online questionnaire on trans health that used a diversified convenience sampling. Cis participants and a small portion of the TNBP sample were recruited through EDADES, a household-based national survey on drug use with a general population random sampling design. Past 12-months and past-month use was assessed for 11 substances. Prevalence of use was calculated for trans women, cis women, trans men, cis men and non-binary people. Adjusted prevalence ratios (aPR) were estimated using Poisson regression models with robust variance, comparing trans women vs. cis women, trans men vs. cis men and non-binary people vs. cis men and women, adjusting for sociodemographic covariables.

**Results:**

Cannabis was the most used substance in all five groups across both time periods. In the past 12 months, poppers were the second most commonly used substance among trans women, while non-medical use tranquilisers ranked second among trans men and non-binary people. Compared with cis peers, aPRs were higher for all substances among trans women and non-binary people and for most among trans men. Overall, the highest aPRs were observed in poppers use, whereas cocaine and cannabis showed smaller gaps.

**Conclusions:**

TNBP show a higher prevalence of substance use compared with their cis peers, particularly women. Public health policies should consider the cultural and social specificities of substance use within TNB communities in addition to structural vulnerabilities.

**Supplementary Information:**

The online version contains supplementary material available at https://doi.org/10.1186/s13011-026-00741-6.

## Background

Transgender and non-binary people (TNB people) —those whose gender identity differs from the sex assigned to them at birth— face a significant burden of adverse health outcomes, including poorer mental health, an increased prevalence of HIV and other sexually transmitted infections, and elevated cardiovascular risk, among others [1–4]. The minority stress theory, conceptualized by Brooks and developed by Meyer [5, 6], and later adapted to trans communities by Hendricks and Testa [7], posits that chronic stress from discrimination and social stigma contributes to these poorer health outcomes, most notably in mental health [5–9]. Furthermore, structural transphobia, which arises from policies, groups and institutions that have historically and continue to deny equal rights to trans communities, contributes to health inequities and acts as a determinant of health [10–12].

Substance use is a potential health-risk behaviour that, similar to patterns observed in sexual minority populations [13], might be presented among a higher proportion of TNB individuals compared with cisgender individuals (those who identify with their sex assigned at birth) [14–17]. It is important to highlight how this difference can be explained by how various substances may carry cultural meanings and community-based dimensions in addition to minority stress theory. This means substance use can be influenced by multiple factors, such as the pursuit of pleasure, self-exploration, identity formation or sociability within specific leisure and social environments and, in some cases, coping-related motives [18, 19].

However, most of the studies published to date share a set of common limitations. The first is that they tend to focus primarily on trans women living in large urban centers and belonging to particularly vulnerable groups —such as those living with HIV, engaged in prevention programs or sex workers [14, 20]— which may lead to an overestimation of substance use and limit the generalizability of their findings. Non-binary individuals remain significantly understudied compared to trans women and men [21]. Additionally, many studies investigate only alcohol [22, 23], combine multiple substances into a single category without disaggregating by substance, and/or sample sizes are modest [19, 24, 25]. Furthermore, studies are primarily focused on the United States (US) or other Anglo-Saxon countries, with limited evidence available from Europe, particularly from Southern European countries [14–17]. Connolly et al., using Global Drug Survey data, are among the few to have analyzed larger European samples, mainly including countries in Central Europe, offering insights through comparisons with cisgender women and across selected substances [26, 27]. Gathering information from diverse regions would enable future comparative analyses that consider the potential impact of trans rights, which vary significantly across countries. Finally, as highlighted by a systematic review, studies comparing substance use between TNB and cis people remain scarce [14]. It is essential to compare the prevalence of substance use in both populations to identify potential differences. Identifying these is important not only because of potential health inequities that might arise if they translate into differences in problematic use or harms, but also because they can inform the design of tailored health interventions and risk-reduction strategies that consider the cultural specificities of TNB communities.

In response to these gaps, the aim of this study is to estimate the past 12-month and past-month prevalence of use of multiple substances among TNB people and to compare these estimates with those of cis populations using large-scale data from Spain.

## Methods

### Study design

We conducted a cross-sectional study pooling data from two surveys on substance use, one targeting TNB population and the other targeting general population.

### Study population

The combined study population included 19,948 individuals aged 15–45 years living in Spain. Most of the TNB sample (*n* = 1468) was obtained from Transaludes survey, a self-administered online questionnaire conducted between October 2023 and March 2024. Transaludes participants were recruited through convenience sampling using a variety of channels including social media, influencers, LGBTIQ+ community organizations, the study website, and QR codes on printed flyers distributed through 39 gender identity units of the national health system and leisure venues frequented by the LGBTIQ+ community in Barcelona.

**Fig. 1:**
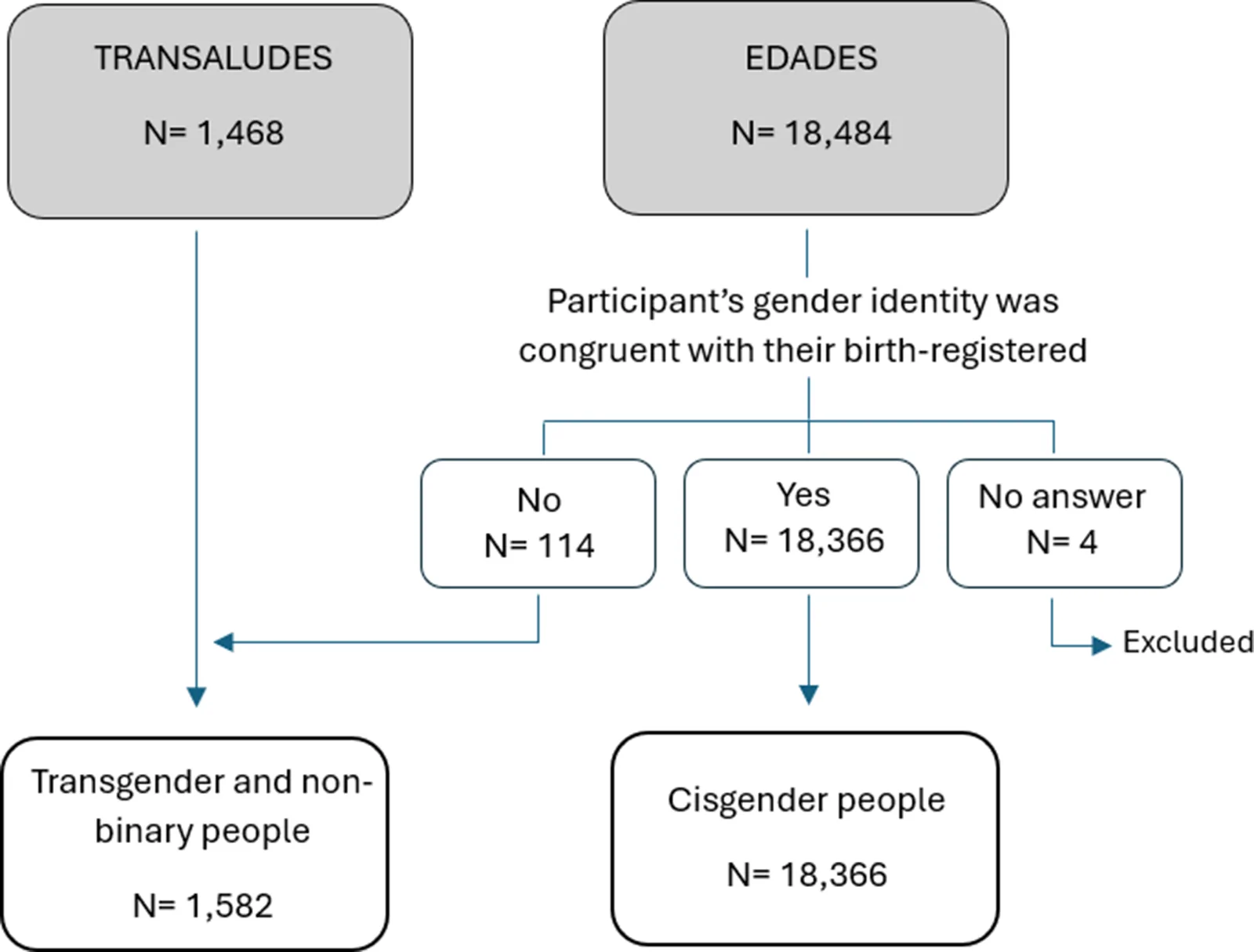
Flow diagram of study participants by survey and gender modality

The general population sample was drawn from EDADES (Encuesta sobre Alcohol y Drogas en España), a household survey focused on alcohol and other drugs conducted between February and June 2024, using a three-stage cluster sampling design without replacement, with census tracts serving as primary sampling units, followed by the selection of households and individuals within households [28]. The questionnaire was administered in two parts: an interviewer-administered section covering sociodemographic and health variables and a self-administered, paper-and-pencil section in which participants reported their drug use. Weights were included based on age, regions, legal gender and size of place of residence. From the 18,484 EDADES respondents, 18,366 identified with their sex assigned at birth (cisgender) and 114 did not (TNB people). Four respondents did not answer the gender identity question and were therefore excluded from the analyses. Given that the small number of TNB respondents in EDADES was too small for meaningful comparisons with the cis sample in that survey —as probability-based sampling tends to underrepresent queer communities [29]— they were combined with Transaludes participants into a single TNB group for analysis. Therefore, the total TNB sample was 1582 and the total cis sample was 18,366 (Fig. 1).

### Measures

Both questionnaires included common questions on sociodemographic characteristics (age, migration status, size of municipality of residence and formal education level), as well as on the use of 11 substances (poppers/volatile inhalants, non-prescription tranquilisers, non-prescription opioids—meaning non-medical use of prescription tranquilisers and opioids, respectively— cannabis, cocaine, amphetamines, methamphetamine, 3,4-methylenedioxymethamphetamine [ecstasy], GHB, hallucinogens, and heroin) across two time periods: past 12-months and past-month. The terminology used to refer to substances in both surveys had minimal differences (Supplementary Table).

### Statistical analysis

First, a frequency distribution of the sociodemographic characteristics of the sample (previously described in Measures) was presented disaggregating by five groups: trans women, cis women, trans men, cis men and non-binary people. Next, for each gender identity group we estimated the prevalence and 95% confidence intervals (95% CIs) of the use of each substance across the two reference time periods.

Adjusted prevalence ratios (aPRs) with 95% CIs were calculated across the two time periods, using Poisson regression models with robust variance. Comparisons were made between trans women and cis women, trans men and cis men, and non-binary people and both cis women and men, accounting for the strong relevance of gender in substance use [30]. The multivariable models that were used to calculate aPRs included as covariates all the sociodemographic variables mentioned in Measures. PRs were not calculated (NC) when the prevalence in one of the comparison groups was zero to avoid undefined results.

The EDADES sampling weights were considered in all calculations. All analyses were performed using STATA 16. A statistical significance level of *p* < 0.05 was set for all calculations. Missing values were treated as such, with no imputations.

## Results

### Sociodemographic characteristics

TNB participants were younger and more likely to reside in large urban areas. Most participants were born in Spain, with a higher proportion of migrants among cis and trans women. The proportion of participants with a university degree ranged from 21.5% to 38.1% across the five groups, with non-binary individuals being the most likely to have a university degree (Table 1).

**Table 1:** Sociodemographic characteristics of the trans women, cis women, trans men, cis men and non-binary people samples

	Trans women	Cis women	Trans men	Cis men	Non-binary
	%	%	%	%	%
Age group (years)					
15–20	21.8	16.3	43.0	17.3	22.3
21–30	41.1	28.5	35.7	28.4	56.1
31–40	24.4	34.7	14.5	34.1	18.3
41–45	12.7	20.5	6.8	20.2	3.3
Migration status					
Born in Spain	80.5	79.5	88.2	83.8	90.1
Migrant	19.5	20.5	11.8	16.2	9.9
Size of municipality residence (inhabitants)					
> 1.000.000	28.4	11.1	17.1	10.9	26.7
> 500.000	8.6	7.9	6.4	7.8	6.4
> 100.000	23.4	21.9	24.3	21.3	26.2
> 50.000	11.7	13.6	13.0	13.1	13
> 10.000	14.8	27.0	22.6	27.6	13.5
< 10.000	13.1	18.5	16.6	19.3	14.2
Formal education level					
None or primary	5.6	4.7	6.2	6.4	1.9
Lowe secondary	15.2	30.9	26.3	33.9	11.9
Upper secondary	51.6	37.2	44.3	38.2	48.1
University	27.6	27.2	23.2	21.5	38.1

Missing values for each variable were: Age group (0%); Migration status (0.3%); Inhabitants (0%); Formal education level (0.66%)

### Substance use prevalence

Overall, the prevalence of substance use was higher among TNB people than among cis individuals. These differences were more pronounced comparing trans women and non-binary people to their cis peers than between trans and cis men. Cannabis was the most used substance in all five groups across both time periods (Table 2).

#### Trans and cis women

The three substances with the highest prevalence of use in the past 12 months among trans women were cannabis (29.6%), poppers (15.6%) and non-prescription tranquilisers (14.4%), whereas among cis women they were cannabis (11.8%), cocaine (1.8%) and non-prescription tranquilisers (1.7%). Regarding past-month use, the most commonly used substances were among trans women cannabis (14.9%), non-prescription tranquilisers (9.1%) and poppers (7.2%), whereas among cis women they were cannabis (9.5%), cocaine (1.0%) and non-prescription tranquilisers (0.8%).

#### Trans and cis men

The most commonly used substance among trans men were cannabis (27.9% in the past 12 months and 16.3% in the past month), non-prescription tranquilisers (13.2% and 6.5%, respectively) and poppers (6.8% and 1.8%, respectively). Meanwhile, among cis men, the most commonly used substances were cannabis (20.5% in the past 12 months and 16.8% in the past month), cocaine (4.5% and 2.6%, respectively) and non-prescription tranquilisers (2.0% and 0.9%, respectively).

#### Non-binary people

The most commonly used substances in this group across the two time periods assessed were cannabis (38.9% in the past 12-months and 22.1% in the past month), non-prescription tranquilisers (19.9% and 10.0%, respectively) and poppers (18.8% and 6.7%, respectively).

**Table 2:** Substance use prevalence by time period among trans women, cis women, trans men, cis men and non-binary people

	Trans women		Cis women		Trans men		Cis men		Non-binary	
Substance	*P*	95% CI	*P*	95% CI	*P*	95% CI	*P*	95% CI	*P*	95% CI
Past 12-month use										
Poppers	15.6	12.0-20.1	0.2	0.2–0.4	6.8	5.1-9.0	0.4	0.3–0.6	18.8	15.9–22.0
Tranquilisers*	14.4	10.9–18.7	1.7	1.5–2.1	13.2	10.7–16.1	2.0	1.6–2.4	19.9	17.0-23.2
Opioids*	4.3	2.5–7.2	0.9	0.7–1.1	2.7	1.7–4.3	0.9	0.7–1.1	4.5	3.2–6.5
Cannabis	29.6	24.8–34.9	11.8	11.0-12.6	27.9	24.5–31.6	20.5	19.5–21.5	38.9	35.2–42.7
Cocaine	11.7	8.6–15.8	1.8	1.5–2.2	2.7	1.7–4.2	4.5	4.0–5.0	7.7	5.8–10.0
Amphetamines	5.3	3.3–8.4	0.4	0.3–0.6	2.4	1.4-4.0	0.6	0.4–0.8	8.5	6.5–10.9
Methamphetamine	3.7	2.1–6.5	0.1	0.0-0.2	0.9	0.4-2.0	0.4	0.2–0.6	2.0	1.2–3.5
Ecstasy	9.4	6.6–13.1	0.6	0.5–0.8	3.6	2.4–5.4	1.4	1.1–1.7	12.1	9.7–14.8
GHB	3.7	2.1–6.5	0.0	0.0-0.1	0.8	0.3–1.8	0.1	0.1–0.2	2.7	1.7–4.2
Hallucinogens	6.1	3.9–9.3	0.5	0.4–0.7	4.1	2.8–5.9	0.9	0.7–1.2	8.6	6.7–11.1
Heroin	1.6	0.6–3.7	0.0	0.0–0.0	0.1	0.0-1.1	0.1	0.0-0.3	0.0	0.0–0.0
Past-month use										
Poppers	7.2	4.8–10.6	0.0	0.0-0.1	1.8	1.0-3.2	0.1	0.0-0.2	6.7	5.0–9.0
Tranquilisers*	9.1	6.4–12.8	0.8	0.6-1.0	6.5	4.8–8.7	0.9	0.6–1.1	10.0	7.9–12.6
Opioids*	1.2	0.5–3.3	0.6	0.4–0.8	1.2	0.6–2.4	0.7	0.5–0.9	1.6	0.8–2.9
Cannabis	14.9	11.4–19.3	9.5	8.8–10.2	16.3	13.6–19.4	16.8	15.9–17.8	22.1	19.1–25.5
Cocaine	6.4	4.2–9.7	1.0	0.8–1.4	1.5	0.8–2.7	2.6	2.2-3.0	2.5	1.5–4.1
Amphetamines	1.6	0.6–3.7	0.1	0.0-0.1	1.2	0.6–2.4	0.2	0.1–0.3	3.9	2.7–5.7
Methamphetamine	0.3	0.0-2.2	0.0^1^	0.0-0.1	0.5	0.1–1.4	0.1	0.0-0.2	0.3	0.1–1.2
Ecstasy	3.4	1.9–6.1	0.2	0.1–0.3	1.8	1.0-3.1	0.5	0.3–0.7	5.5	4.0-7.5
GHB	0.9	0.3–2.9	0.0	0.0–0.0	0.0	0.0–0.0	0.0	0.0–0.0	0.6	0.2–1.7
Hallucinogens	2.8	1.5–5.3	0.1	0.1–0.2	1.8	1.0-3.2	0.3	0.2–0.5	3.4	2.3–5.2
Heroin	1.2	0.4–3.3	0.0	0.0–0.0	0.1	0.0-1.1	0.1	0.0-0.2	0.0	0.0–0.0

P: prevalence; 95% CI: 95% confident interval; NC: not calculated (as at least one of the prevalence was zero). ^1^0.04.*non-medical use

#### Adjusted prevalence ratios

After adjusting for the sociodemographic variables (age, migration status, size of municipality of residence and formal education level), the following results were obtained (Table 3):

#### Trans and cis women

All estimates indicated a significantly higher prevalence of substance use among trans women, except for non-prescription opioids in the past-month. The highest aPRs for past 12-month use were observed for poppers (50.9), methamphetamine (44.6) and amphetamines along with ecstasy (12.5). For past-month use, the highest aPRs were observed for hallucinogens (18.2), ecstasy (18.0) and amphetamines (16.5). The lowest aPRs were observed for cannabis across both time periods (2.3 for past 12-month use and 1.5 for past-month use).

#### Trans and cis men

For most substances, substance use was significantly higher among trans men, although the magnitude of the differences was generally smaller than that observed in women. The highest aPRs were observed for poppers (9.3 for the past 12-months and 14.4 for the past-month), non-prescription tranquilisers (6.6 and 7.3, respectively) and amphetamines (3.9 and 6.9 respectively). Trans men showed a lower prevalence of cocaine use for the past 12 months, although the association was not statistically significant.

#### Non-binary people

For the aPRs comparing with cis women, all indicated significantly higher consumption among non-binary individuals, except for methamphetamine in the past month. The highest aPRs for past 12-month were observed for poppers (54.0), amphetamines (19.2) and methamphetamine (17.0); for past-month use: amphetamines (43.4), hallucinogens (24.4) and ecstasy (21.6).

For the aPRs comparing with cis men, all indicated significantly higher consumption among non-binary individuals, except for cocaine and methamphetamine for past-month use. The three highest aPRs for past 12-month use were observed for poppers (27.2), GHB (15.4) and amphetamines (13.7); for past-month use for poppers (58.3), amphetamines (18.3) and non-prescription tranquilisers (11.9).

**Table 3:** Adjusted prevalence ratios for substance use by time period comparing trans and cis women, trans and cis men, and non-binary individuals with cis women and men^†^

	Trans women/ cis women		Trans men/ Cis men		Non-binary/ Cis women		Non-binary/ cis men	
Substance	aPR	95% CI	aPR	95% CI	aPR	IC 95%	aPR	95% CI
Past 12-month use								
Poppers	50.9	28.8–89.8	9.3	5.9–14.6	54.0	31.3–93.3	27.2	17.5–42.3
Tranquilisers*	9.2	6.5–13.1	6.6	4.9-9.0	12.0	9.2–15.9	10.0	7.6–13.3
Opioids*	4.7	2.6–8.4	3.2	1.8–5.7	5.1	3.2–8.3	4.7	2.9–7.7
Cannabis	2.3	1.9–2.8	1.3	1.1–1.4	2.8	2.4–3.2	1.8	1.6-2.0
Cocaine	6.0	4.1–8.8	0.7	0.4–1.1	3.6	2.5–5.3	1.9	1.4–2.7
Amphetamines	12.5	6.2–25.0	3.9	2.1–7.2	19.2	10.7–34.8	13.7	8.8–21.3
Methamphetamine	44.6	16.5-120.7	2.2	0.8–6.1	17.0	5.5–52.5	5.9	2.5–13.9
Ecstasy	12.5	7.6–20.7	2.0	1.3–3.3	13.9	9.2–21.1	6.5	4.6–9.1
GHB	NC	NC	3.3	1.0^1^–10.7	NC	NC	15.4	6.5–36.6
Hallucinogens	11.1	6.3–19.6	3.6	2.2–5.9	14.1	8.6–22.9	7.8	5.1–12.0
Heroin	NC	NC	1.2	0.1–13.9	NC	NC	NC	NC
Past-month use								
Poppers	NC	NC	14.4	6.0-34.2	NC	NC	58.3	24.4-139.4
Tranquilisers*	11.5	7.0-18.7	7.3	4.6–11.6	12.4	8.1–19.2	11.9	7.6–18.7
Opioids*	2.0	0.7–5.8	1.9	0.9–4.3	2.5	1.2–5.3	2.2	1.0^4^–4.5
Cannabis	1.5	1.1–1.9	0.9	0.8–1.1	2.0	1.7–2.4	1.3	1.1–1.5
Cocaine	5.6	3.3–9.5	0.7	0.3–1.3	2.2	1.2-4.0	1.1	0.6–1.9
Amphetamines	16.5	5.3–51.1	6.9	2.7–17.2	43.3	17.9-105.1	18.3	8.3–40.5
Methamphetamine	7.2	1.1–46.2	6.3	1.0^2^–39.6	8.1	1.0^3^–68.3	3.9	0.8–18.0
Ecstasy	18.0	7.7–42.3	2.7	1.3–5.6	21.6	10.8–43.1	7.6	4.3–13.4
GHB	NC	NC	NC	NC	NC	NC	NC	NC
Hallucinogens	18.2	6.9–47.8	5.0	2.3–10.9	24.4	9.1–65.3	9.9	4.7–20.9
Heroin	NC	NC	2.8	0.2–40.6	NC	NC	NC	NC

aPR: adjusted prevalence ratios; 95% CI: 95% confident interval; NC: not calculated (as at least one of the prevalence was zero). ^†^Adjusted by age, migratory status, size of municipality of residence and education level. ^1^1.03; ^2^1.005; ^3^0.96; ^4^1.04. *non-medical use

## Discussion

### Main findings

Overall, our study, one of the first in Europe to estimate and compare the prevalence of a wide range of substances in a large sample of TNB and cis people, found a higher prevalence of substance use among the TNB population compared to their cis peers. These differences were particularly pronounced among trans women and non-binary individuals when compared with cis women. This pattern appeared to be largely driven by the high prevalence of substance use among trans women and the comparatively low prevalence observed among cis women.

Cannabis was the most commonly used substance in all five groups across both time periods and was among those with the smallest prevalence differences between TNB and cis participants. Cocaine was the only substance (with cannabis for the past-month) for which prevalence was higher among cis people (cis men) when comparing to trans (trans men), although this difference did not reach statistical significance. The largest differences in prevalence were observed for poppers, methamphetamine, amphetamines, ecstasy, hallucinogens and non-prescription tranquilisers, with substantially higher prevalence estimates among trans participants. Some comparisons could not be performed because the prevalence of certain substances (poppers, GHB and heroin) was zero in one or more cis groups. Nevertheless, the observed prevalence differences were considerable.

### Findings in context

In Southern Europe, current literature on substance use among trans population remains scarce. Several studies focus exclusively on alcohol, cannabis and cocaine, aggregate data across different substances, or rely on small samples [31–35]. One study included a large sample of European participants, although its focus was primarily centered on Central European countries [26]. One study reported cannabis use prevalence rates similar to those presented here [33], while other suggested no differences in use between trans and cis people [32]; but sometimes comparability is limited due to the aforementioned methodological limitations of previous research such as recruiting participants only in gender affirming care centers, not adjusting for sociodemographic differences between the samples, or not differentiating specific substances.

In the US and other non-European Anglophone countries, there is a larger body of literature on the subject. A systematic review and a meta-analysis (both based primarily or exclusively on studies conducted in the US) found higher prevalence rates of substance use among trans individuals [14, 15]. The meta-analysis reported significant differences for lifetime use and current use of specific substances, including cocaine, amphetamines, methamphetamine, ecstasy, and cannabis, findings consistent with our study. Another large-scale study conducted in 2017 found a higher prevalence of substance use disorders among trans population compared to cis individuals [36]. When focusing on young people, both a scoping review and a systematic review reported findings consistent with ours, showing higher prevalence of the use of several substances among trans individuals, including methamphetamine, ecstasy, poppers and others, and interpreting such patterns through the minority stress theory [16, 17]. The findings in our study suggest that the differences documented in the US might be similar in other contexts despite differences in drug markets, legal frameworks, and trans rights protections [37–39].

### Interpretation and contextualization of the results

Although substances which could be more likely used as a coping mechanism (e.g., tranquillisers —even though its use is not only for this reason—) showed differences in consumption between TNB and cis individuals, the largest differences were observed for substances more culturally associated with the LGBTIQ+ community, such as poppers and substances sometimes associated with chemsex [40].

Minority stress and discrimination are sometimes cited to explain reasons behind differences in drug use between queer and cis-heterosexual communities [9]. While this framework provides an important perspective, it is worth noting that it can be simplistic, stigmatizing or even pathologizing, as it might overlook the role of culture in substance use, which shapes patterns and meanings of use, as it does in other social groups [13, 41]. Previous research has shown that substance use can be relevant in identity formation, expression and social bonds within queer communities [42]. Therefore, differences in substance use should not be understood solely as coping mechanism. Motivations —which were not explored in this paper— related to pleasure, culture and sociability are also relevant [19, 43, 44].

This highlights the importance of developing harm reduction strategies and social policies that consider cultural specificities from non-stigmatizing perspectives. Given that cultural and social contexts may partly explain the observed differences in substance use, public health responses should incorporate approaches that engage with queer social environments and community-based settings, which are scarce to date [45]. This involves designing social policies and harm reduction initiatives that are not exclusively grounded in dominant cis-heteronormative frameworks but instead acknowledge substance use patterns that may emerge within specific queer social or sexual networks, in line with principles of equity and social justice, following previous interventions [46]. Nonetheless, it is important to highlight that coping mechanisms linked to substance use, explained by minority stress, transphobia, and structural barriers, are extensively documented and must be considered [16, 17]. Moreover, the widely described lack of cultural competence and training on trans and non-binary health among healthcare professionals can generate distrust and pose a barrier to accessing healthcare services, hindering TNB individuals from seeking support in the case of problematic substance use [47, 48]. From this perspective, as presented by previous evidence [49], campaigns against social transphobia and discrimination, as well as specialized training for healthcare professionals on the specific motivations underlying TNB substance use, also constitute efforts to reduce potential health inequities related to substance use.

Future research should assess which factors explain different aspects of these differences by applying an intersectional approach and considering discrimination but also cultural factors and the pursuit of pleasure. It is important to recognize the heterogeneity of consumption patterns within the TNB population, which may vary according to gender identity, sexual orientation, age, stage of gender affirmation, or socioeconomic status, as highlighted in three recent reviews [14, 16, 17]. This underscores that need for intersectional analyses that examine how these factors interact with structural determinants and cultural contexts to influence substance use patterns.

### Strengths and limitations

Certain methodological limitations should be acknowledged, primarily regarding the differences in recruitment methods between the two surveys, which may have introduced different forms of selection bias. The Transaludes sample was recruited through community-based and online strategies, whereas the EDADES sample was derived from a household-based survey. Community recruitment may overrepresent individuals who are more socially connected, while household sampling may underrepresent marginalized individuals who are less reachable through residential addresses. Nevertheless, as previously discussed in minority health research [29, 50, 51], non-probability sampling is often necessary to adequately study small and underrepresented populations such as TNB individuals, who are typically insufficiently represented in general population surveys to ensure adequate statistical power. Furthermore, the adjusted analyses took into account several sociodemographic characteristics available in both surveys, reducing the potential influence of differences in these variables between samples. However, other potentially relevant characteristics, such as sexual orientation, were not available in both surveys and therefore could not be included in the analyses. Additionally, participants’ ages were restricted to a range of 15 to 45 years; this specific range is a priority target in public health strategies related to substance use for its higher prevalence of use and use initiation [28, 52]. Whether differences in substance use translate into differences in problematic substance use could not be analyzed as EDADES survey did not measure problematic substance use. Finally, the study did not estimate or compare the prevalence of alcohol and tobacco use; instead, it focused on the use of illegal substances in Spain and non-medical use tranquilisers/opioids. Although this limits comparability with part of the existing literature on the subject, the study provides evidence on less frequently examined substances such as poppers, ecstasy, amphetamines, GHB, non-medical use tranquilisers and others.

Despite these limitations, the study has substantial strengths that address critical gaps identified in previous reviews. One important strength of this study is the focused analysis of substance use among trans men, utilizing cisgender men as a formal statistical reference group. This comparative approach addresses a notable gap in the literature, where such systematic comparisons are infrequent. Furthermore, the study explicitly incorporates non-binary people, a population that remains largely overlooked in scientific research [53], thereby providing a more comprehensive and inclusive overview. The large sample size allows for the calculation of estimates with narrow 95% CI, which is highly valuable for public health strategies. Adjustment for key sociodemographic variables enhances the robustness of comparisons. Finally, both surveys were self-administered, an important aspect in substance use research, as this may reduce social desirability bias. However, as with all self-reported measures, the finding remains susceptible to recall errors and potential underreporting or overreporting of substance use.

## Conclusions

The TNB population shows a higher prevalence of substance use compared with their cis peers, particularly women. Cannabis is the most frequently consumed substance, followed by poppers in trans women and non-prescription tranquilisers in trans men and non-binary people (for the past 12-months). The most pronounced differences between TNB and cis populations are observed in poppers use, whereas cocaine and cannabis show smaller gaps.

These findings, consistent with international evidence, underscore the need for targeted public health policies and specialized healthcare training that consider the cultural and social specificities of substance use within the TNB population. This should include strengthening trans-affirming services across primary care, specialized healthcare (including mental health and addiction services), and community-based settings. In addition, healthcare professionals should receive training to better understand the specific needs of TNB populations and the motivations underlying substance use, enabling the development of interventions that address the structural and social determinants of these patterns. Further research is also needed to identify the factors contributing to higher levels of substance use among trans women and non-binary individuals compared with trans men, taking into account multiple axes of inequality within an intersectional framework. Such measures should promote respectful and non-stigmatizing interventions, such as harm reduction strategies and social policies, that acknowledge gender self-determination, while implementing strategies to combat discrimination and transphobia.

## Supplementary Information

Below is the link to the electronic supplementary material.

Supplementary Material 1

## Data Availability

The datasets used and/or analyzed during the current study are available from the corresponding author on reasonable request.

## Abbreviations

95% CI: 95% confident interval
GHB: Gamma hydroxybutyrate
HIV: Human Immunodeficiency Virus
LGBTIQ+: Lesbian, gay, bisexual, trans, intersexual, queer plus
aPR(s): Adjusted prevalence ratio(s)
QR: “Quick Response” code
TNB(P): Trans and non-binary (people)
US: United States
