# Supplementary material for: Differences in substance use among trans, non-binary and cis people in Spain: a cross-sectional study
Source: Subst Abuse Treat Prev Policy. 2026 Jul 8;21:63. doi: 10.1186/s13011-026-00741-6 (PMC13640274; doi:10.1186/s13011-026-00741-6)
Supplement: Supplementary file 1 [file 13011_2026_741_MOESM1_ESM.docx]

| Substance | Transaludes | EDADES |
| --- | --- | --- |
| Poppers | **Poppers** or volatile inhalants (glue, glue, solvent, nitrites, gasoline) | **Volatile inhalants** (**poppers**, glue, glue, solvent, nitrites, gasoline) |
| Tranquilisers* | **Non-prescription tranquilizers** or for non-medical use  (Valium®, Lexatin®, Trankimazin®, etc.) | **Non-prescription or non-medical tranquilizers/sedatives** (medicines to calm nerves or anxiety) and/or **non-prescription or non-medical sleep aids** (sleep aids). |
| Opioids* | **Non-prescription or non-medical opioids** (opium, tramadol, codeine, fentanyl, methadone, morphine, etc.) | **Non-prescription or non-medical opioid analgesics** (morphine-derived pain relievers). |
| Cannabis | **Cannabis** or synthetic cannabinoids (marijuana, hashish, joints, synthetic marijuana, spice, K2…) | **Cannabis, marijuana, or hashish** (hash oil, chocolate, joint, cost, weed) |
| Cocaine | Powdered or crack **cocaine**  (farlopa, base, basuco, etc) | **Cocaine in powder form** (cocaine, parakeet, farlopa, cocaine hydrochloride) +  **cocaine in base form** (base, cooked cocaine, basuco, bowls, crack, rock) |
| Amphetamines | **Amphetamine/speed** | **Amphetamines or speed** (anfetas) |
| Methamphetamine | **Methamphetamine** (tina,  meth, T) | **Methamphetamine** (meth, ice, tina) |
| Ecstasy** | **Ecstasy** o MDMA in its pills form (pills, lollipops) or in its crystalline or powder form or powder form (M, crystal) | **Ecstasy or other synthetics drugs** (pills, lollipops, MDMA) |
| GHB | **GHB**/GBL (G, canister, liquid ecstasy) | **GHB** (liquid ecstasy, gamma-hydroxybutyrate, x-liquid, gamma-OH) |
| Hallucinogens | **Hallucinogens** (LSD, acid, tripis, magic mushrooms) + **Ketamine** (K, keta, kei) | **Hallucinogens** (lsd, acid, tripi, magic mushrooms, ketamine, special-k, ketolar, imalgene) |
| Heroin | **Heroin** (caballo, jaco…) | **Heroin** (caballo, jaco) |
| *non-medical use; **3,4-methylenedioxymethamphetamine; GHB: gamma hydroxybutyrate | | |

**Supplementary Table S1**. Wording of the drug use questions in both surveys.
